# The relationship between time anxiety and college students’ sleep quality: the mediating role of irrational procrastination and the moderating effect of physical activity

**DOI:** 10.3389/fpsyg.2024.1410746

**Published:** 2024-07-04

**Authors:** Zhe Sun, Xinchao Gao, Penghui Ren

**Affiliations:** ^1^Physical Education Department, Northeastern University, Shenyang, China; ^2^Physical Education Department, Yuncheng Vocational and Technical University, Yuncheng, China

**Keywords:** time anxiety, sleep quality, irrational procrastination, physical activity, college students

## Abstract

**Background:**

Poor sleep quality has become one of the most pressing public issues among Chinese college students, with an increasing incidence rate in recent years. Although some studies showed that anxiety is related to sleep quality, the relationship between time anxiety (which is a more concrete manifestation of anxiety in the temporal dimension) and sleep quality, as well as its potential mechanisms, still requires further investigation and analysis. This study aimed to explore the relationship between time anxiety and sleep quality among college students, and to examine the mediating role of irrational procrastination and the moderating effect of physical activity.

**Methodology:**

A cross-sectional study was conducted with 1,137 participants recruited from four universities in eastern, western, and central China. They completed a questionnaire survey on time anxiety, irrational procrastination, physical activity, and sleep quality. Data analysis was performed using SPSS 26.0 and PROCESS 3.3.

**Results:**

Time anxiety had a significant positive impact on sleep quality (*β* = 0.28, *t* = 9.95, *p* < 0.001). Irrational procrastination played a mediating role between time anxiety and college students’ sleep quality, the effect value was 0.05, and the intermediary effect accounted for 19.26%. Physical activity moderated the direct effect of time anxiety on college students’ sleep quality (*β* = −0.08, *t* = −2.98, *p* < 0.01), and moderated the second half path of irrational procrastination mediation model (*β* = −0.06, *t* = −2.12, *p* < 0.05).

**Conclusion:**

Higher levels of time anxiety are associated with poorer sleep quality among college students. Time anxiety not only directly affects college students’ sleep quality, but also indirectly affects it through irrational procrastination. Conducting physical activities can mitigate the impact of time anxiety and irrational procrastination on college students’ sleep quality.

## 1 Introduction

Sleeping is a requisite process for human life activities, serving as an essential guarantee for normal body functions and good quality of life (Zhu and Huang, 2022). As the social economy grows by leaps and bounds, various stress-inducing events have emerged, making the living environment of college students increasingly complicated, thereby leading to the increasingly prominent sleep quality issues among college students. Survey results showed that about 25% of Chinese college students experienced sleep quality problems (You et al., 2021), which might not only affect their academic performance (Maheshwari and Shaukat, 2019), but also give rise to adverse health behaviors such as smoking and excessive drinking (Tao et al., 2017), and even increase the risk of suicidal intention (Sun et al., 2021). Numerous studies have proved that anxiety is a significant factor leading to sleep disorders (Liu et al., 2021). What’s even worse, “anxiety” has become a highly representative collective mentality in today’s society (Zhou, 2014), with this collective sense of anxiety being especially prominent in relation to time. Therefore, “time anxiety” has undoubtedly become a new focus in exploring the relationship between anxiety and sleep quality.

Generally speaking, individuals often find themselves facing recurring conflicts between their desire for effective time management and feelings of frustration (Huang and Sang, 2018). This is frequently compounded by worries, concerns, and anxieties about the passage of time. When this form of anxiety becomes a common symptom of social groups, it transcends the realm of ordinary psychology to become a group syndrome with contemporary structural features (Li, 2023). Recent studies indicated that young people with high time anxiety levels often proactively stay up late, seeking to regain control over time and reconstruct meaningful personal discretionary time, thus alleviating feelings of anxiety. However, such “distorted” behavior not only fails to address the root cause of time anxiety (Gu, 2023), but also tends to worsen sleep quality issues (Adelantado-Renau et al., 2019). For young college students who are in the emerging adulthood phase, they often experience tremendous changes in cognition, emotions, roles, and behaviors (Peng et al., 2012). This period is marked by a certain level of ambiguity in self-identification and self-concept (Ar-yuwat et al., 2013). Their subjective experiences with time anxiety, time management tendencies and career aspirations are notably sensitive to time issues. Therefore, investigating the relationship between time anxiety and sleep quality among college students could offer fresh insights into improving their mental health and sleep quality.

### 1.1 Time anxiety and sleep quality

**Figure 1:**
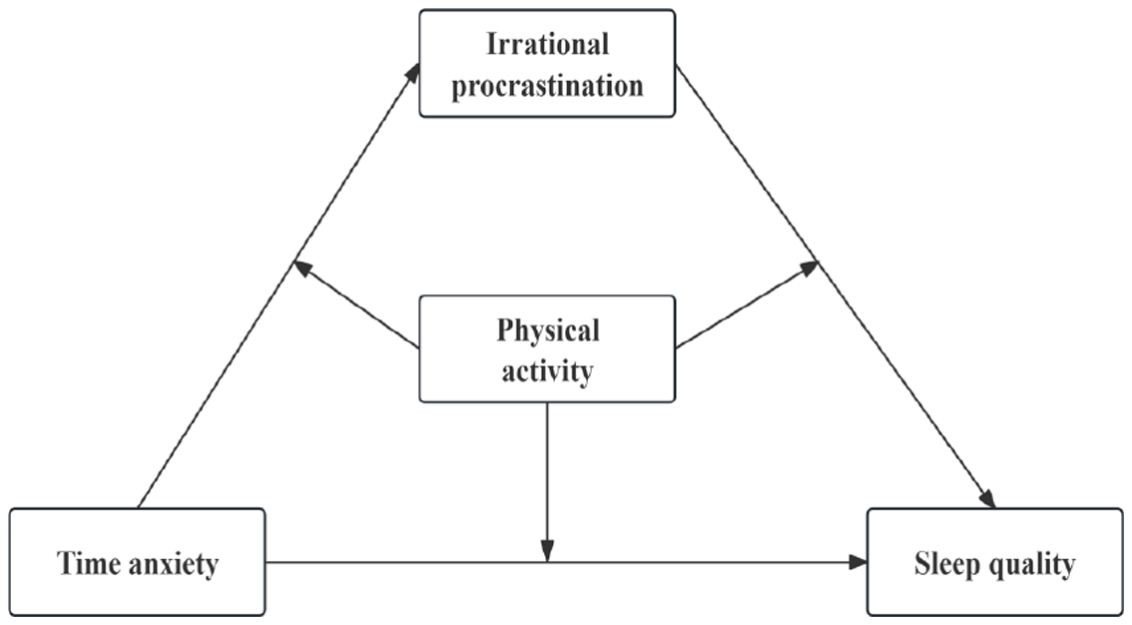
Hypothetical theory model.

The concept of time anxiety, first proposed by Tillich, suggests that time anxiety stems from the developmental process of existence to non-existence, “Time anxiety stems from the alienation of the individual’s perception of his or her own self-existence, i.e., the experience of non-existence that arises from the inner level as a wasted life and a waste of time.” (Tillich, 2000). Based on this, scholars articulated a more tangible definition of time anxiety, considering it as one of the individual’s subjective experiences, characterized by a state of tension that emphasizes proper planning, full utilization of time, avoidance of wasting time, as well as the negative behaviors and tendencies that arise from it (Chen, 2013). Generally speaking, the “time” that triggers time anxiety is multifaceted rather than one-dimensional. The “time” can serve as both a background framework constraining individual behavior and a resource available for individual use (Torre, 2007). Relevant studies indicated a correlation between the scarcity of time resources and mental health outcomes (DeSousa et al., 2020). When time resources are limited, it heightens individual anxiety, consequently influencing their behavioral patterns (Berenskoetter, 2020). Recent studies have shown that moderate time anxiety is beneficial for personal growth, but overly reinforced time anxiety can distort one’s perception of time and space, leads to subject alienation (Chen and Xu, 2022), further exacerbate individuals’ psychological burden, impairs their ability to effectively cope with time pressure, and lead to more negative and passive behavior patterns (Du and Chen, 2023). This, in turn, affects their rational and autonomous behaviors, such as working overtime voluntarily and reducing rest time (Huang and Sang, 2018). Additionally, according to the Sleep Disturbance Process Theory, negative psychological emotions caused by excessive emotional arousal can disturb individuals’ normal sleep process, leading to poor sleep quality (Lundh and Broman, 2000). Negative emotions caused by improper time management and other factors can negatively predict individual sleep quality, and this has been verified by many scholars. For instance, studies found that stress emotions caused by improper time management can affect the overall sleep quality of college students (Knowlden and Naher, 2023). Scovelle et al. (2023) also found that time pressure had a negative impact on sleep quality. All in all, existing studies confirmed that negative emotions related to time can adversely affect individual sleep quality. Through literature review, it was found that academic studies primarily focus on negative emotions triggered by inefficient time management mechanisms and their correlation with sleep quality. However, few studies have explicitly investigated the relationship between time anxiety as an independent variable and sleep quality among college students. In an era marked by terms like “accelerating society” and “time famine,” in an era marked by terms like “accelerating society” and “time famine,” it remains to be thoroughly explored whether time anxiety, as an important issue reflecting societal development, affects the sleep quality of college students as a unique group. In view of this, Hypothesis 1 was proposed: Time anxiety may affect the sleep quality of college students (Figure 1).

### 1.2 The mediating role of irrational procrastination

Procrastination refers to the behaviors where individuals, despite foreseeing negative consequences, delay or postpone the necessary tasks to achieve their goals. This tendency is considered a stable trait or behavioral inclination (Lay, 1986). Irrational procrastination, however, refers to maladaptive behaviors stemming from failures in self-regulation, typically accompanied by negative emotions and psychological states (Steel, 2010). Notably, irrational procrastination places a greater emphasis on the shifts in individual psychological factors, compared to the general concept of procrastination. Given this distinction, this study adopts irrational procrastination as a mediating variable to more accurately assess whether procrastination among college students serves as a mediator between time anxiety and sleep quality from the perspective of psychological health. Previous studies have shown that irrational procrastination is associated with increased symptoms of depression and anxiety (Ferrari et al., 1995), while negative psychological emotions may influence the irrational procrastination behaviors of individuals to some extent. According to the Theory of Self-regulation Failure, irrational procrastination is a result of individuals’ failure in self-regulation, and it can be triggered by fatigue, resource depletion, and reduced self-regulation ability (Pychyl and Flett, 2012). From the perspective of the concept of time anxiety, there is a significant negative correlation between time management tendency and anxiety (Wu et al., 2014), and this psychological emotion has a significant negative impact on individuals’ procrastination (Zheng et al., 2018). In other words, when an individual’s time management resources are depleted, it often triggers anxiety, potentially resulting in irrational procrastination. In addition, according to the Procrastination Health Model (Sirois et al., 2003), the propensity for procrastination is associated with negative health outcomes, such as insomnia symptoms (Sirois, 2007; Hairston and Shpitalni, 2016). Combining Steel’s perspective, irrational procrastination, as a maladaptive behavior resulting from self-regulation failure, is an important factor affecting health (Steel, 2010). Relevant empirical studies also indicated a significant negative correlation between negative procrastination behaviors (also known as irrational procrastination) and healthy behaviors (Ni et al., 2016). Therefore, we hypothesized that irrational procrastination may also be a key factor that influences individual sleep quality. In conclusion, based on relevant theories and research, Hypothesis 2 was proposed: Irrational procrastination mediates the relationship between time anxiety and sleep quality among college students (Figure 1).

### 1.3 The moderating effect of physical activity

Physical activity refers to the process of using various sports methods and combining natural forces to exercise the body, with the aim of improving health and physical fitness (Xi, 2004). As a positive and healthy lifestyle choice, physical activity not only promotes individuals’ mental health, but also influences individual behavior habits and patterns (Wei, 2023). According to the integrated model of sports performance, the positive psychological activities (such as cognitive, emotional, and physiological experiences) generated during sports have a protective effect on individual physical and mental health, and can effectively mitigate unhealthy behaviors to some extent (Liu and Shi, 2009). Studies suggested that individuals frequently involved in physical activities tend to adopt scientific exercise strategies to improve their adaptability and emotional management abilities when faced with negative emotions and other factors (San Román-Mata et al., 2020), in order to dissolve their negative emotional experiences and enhance positive emotional experiences (Li et al., 2015). These emotional changes help individuals regulate physiological mechanisms related to sleep, thereby improving sleep quality (Yang and Li, 2023). From the perspective of exercise psychology, the psychological structure of sports is highly compatible with that of mindfulness, possessing significant emotional regulation and self-acceptance abilities (Xu, 2020). It can enhance one’s psychological capital to address negative events and experiences (Lindsay and Creswell, 2017), promote self-awareness of current cognition, thinking, and emotions (Lutz et al., 2016), and reduce distractions and attention issues (Verhaeghen, 2021), thereby enhancing individual self-control and reducing the likelihood of self-regulation failure (Leyland et al., 2019). Additionally, according to the theory of planned behavior, individuals’ behavioral intention can directly determine their behavior (Ajzen, 1991). Related studies showed that individuals who regularly engage in physical activities tend to have higher self-control abilities (Finley and Brandon, 2019), which help mitigate the impact of negative automatic behaviors and other factors (Baumeister et al., 2007), thus diminishing the adverse consequences caused by procrastination (Liu, 2020). In light of the aforementioned theories and empirical studies, Hypothesis 3 was proposed: Physical activity plays a moderating role in the relationship between time anxiety and sleep quality, time anxiety and irrational procrastination, and irrational procrastination and sleep quality (Figure 1).

In summary, this study constructed a moderated mediation model by integrating multiple theories and previous research. This model was used to explore the relationship between time anxiety and college students’ sleep quality, with a specific focus on the mediating and moderating effects of irrational procrastination and physical activity, in order to shed light on the underlying mechanisms of college students’ sleep quality, reveal various issues in their daily academic lives, and provide theoretical insights and practical guidance for developing targeted intervention strategies in the future. The hypothetical theory model is illustrated in Figure 1.

## 2 Methodology

### 2.1 Participants

A questionnaire survey was conducted using the convenience sampling method. A total of 1,357 college students were selected from four universities located in the eastern, western and central regions of China to participate in the questionnaire survey. Among them, 1,137 valid questionnaires were collected, resulting in a valid response rate of 82.69%. Among the respondents, there were 708 males (62.27%) and 429 females (37.73%); in terms of age, 108 respondents were 18 and below (9.50%), 271 were 19 years old (23.83%), 366 were 20 years old (32.19%), 200 were 21 years old (17.59%), 117 were 22 years old (10.29%), and 75 were 23 years old and above (6.60%); regarding their field of study, 273 were majored in humanities (24.01%) and 864 were majored in science and engineering (75.99%).

Before the survey, the subjects were clearly informed of the purpose of the study and the voluntary nature of participation in the questionnaire, and the anonymity and confidentiality of the data collection were emphasized, informing them that the data collected would be used only for scientific research and that no information would be disclosed. Data collection was carried out on the basis of obtaining the subjects’ permission and signing an informed consent form. Finally, the studies involving humans were approved by the Science and Technology Division of Yuncheng Vocational and Technical University, and they were conducted in accordance with local legislation and institutional requirements.

### 2.2 Measurements

#### 2.2.1 Time anxiety scale

The time anxiety scale developed by Usunier and Pierre (1994) was adopted to measure the subjects’ anxiety towards time control or discomfort. This scale consists of four items, which are rated on a 7-point scale ranging from 1 (totally disagree) to 7 (totally agree). Higher scores indicate a higher level of time anxiety of individuals. In this study, the Cronbach’s *α* coefficient for the time anxiety scale was calculated as 0.69.

#### 2.2.2 Irrational procrastination scale

With reference to the Irrational Procrastination Scale revised by Lian et al. (2018), the questionnaire was used to assess participants’ irrational procrastination behavior. The scale consists of 9 items rated on a 5-point scale, ranging from 1 (totally disagree) to 5 (totally agree). The scores of all items are added together to get each survey respondent’s total score. Higher scores indicate a higher frequency of irrational procrastination behavior. In this study, the Cronbach’s *α* coefficient for the scale was 0.78.

#### 2.2.3 Physical activity level scale

The quantification of physical activity is conducted using the “Physical Activity Level Scale” developed by Liang (1994). This scale assesses physical activity levels from three dimensions: intensity, frequency, and duration of exercise, which are then used to categorize physical activities into large, medium, and small levels. The physical activity level score is calculated as Exercise Intensity × (Exercise Duration – 1) × Exercise Frequency, using a Likert 5-point scoring method, with scores ranging from 1 to 5 and a total possible score of 100. A total score of ≤19 points indicates a low level of physical activity, 20–42 points indicate a moderate level of physical activity, and a score of ≥43 points indicates a high level of physical activity. The Cronbach’s *α* coefficient for the Physical Activity Level Scale measured in this study is 0.61.

#### 2.2.4 Pittsburgh sleep quality index

Sleep quality was measured by using the Pittsburgh Sleep Quality Index compiled by Buysse et al. (1989). The scale consists of 18 items, including 7 factors: sleep quality, sleep latency, sleep duration, sleep efficiency, sleep disturbance, and daytime dysfunction. The scale employs a 4-point scoring method, with scores ranging from 0 to 3 based on the degree of sleep quality. The total score is obtained by summing all the items, with higher scores indicating poorer sleep quality. In this study, the Cronbach’s *α* coefficient for the PSQI was 0.84.

### 2.3 Research procedure

In this study, all respondents read the informed consent form and agreed to voluntarily participate in the survey. Secondly, all respondents completed the questionnaire in a quiet indoor environment. Finally, data for this study were selected by removing invalid questionnaires that violated response rules, had insufficient completion time, or contained missing responses.

Data analysis in this study was performed using SPSS 26.0. Descriptive statistics were used to describe the demographic information of the participants. Pearson correlation analysis was used to assess the relationships between variables. The moderating mediation model was constructed using the PROCESS3.3 macro program, which utilizes bootstrap resampling. The 95% confidence interval (CI) was calculated based on 5,000 bootstrap samples to estimate the mediating and moderating effects. Results were considered statistically significant if the confidence interval did not include zero.

## 3 Results and analysis

### 3.1 Common method deviation test

Harman’s single factor test was adopted. All subjects related to the four variables (time anxiety, irrational procrastination, physical activity and sleep quality) were included in the exploratory factor analysis. Seven factors with eigenvalues greater than 1 were calculated, which explained 58.374% of the variation. The explained variation of the first factor was 21.45%, which was less than the critical value of 40%. This indicated that there was no obvious common method bias in the survey process.

### 3.2 Descriptive statistics and correlation analysis

The results of the descriptive statistics and correlation analysis (Table 1) indicated that time anxiety is significantly positively correlated with irrational procrastination (*r* = 0.49, *p* < 0.001) and sleep quality (*r* = 0.27, *p* < 0.001). Irrational procrastination is also significantly positively correlated with sleep quality (*r* = 0.22, *p* < 0.001). Physical activity is significantly negatively correlated with time anxiety (*r* = −0.08, *p* < 0.01), irrational procrastination (*r* = −0.12, *p* < 0.01), and sleep quality (*r* = −0.29, *p* < 0.001). Based on the degree of correlation between these variables, it suggests the feasibility of conducting further statistical analysis.

**Table 1:** Standard deviation, mean and correlation matrix of each variable.

Variable	*M* ± *SD*	Time anxiety	Irrational procrastination	Physical activity	Sleep quality
Time anxiety	12.27 ± 2.51	1			
Irrational procrastination	26.38 ± 3.40	0.49***	1		
Physical activity	24.39 ± 21.41	−0.08**	−0.12**	1	
Sleep quality	6.68 ± 3.25	0.27***	0.22***	−0.29***	1

*p < 0.05, **p < 0.01, ***p < 0.001. This is also applicable to the table below.

### 3.3 Analysis of mediating effect test

The mediating effect results of irrational procrastination behavior are shown in Table 2. Mediation effect was tested for the relationship among time anxiety, irrational procrastination and sleep quality by using the PROCESS plug-in compiled by Hayes and using model 4, while controlling for gender, age, and major. In the absence of intermediary variables, time anxiety exerted a significant positive impact on sleep quality (*β =* 0.28, *t =* 9.95, *p <* 0.001), it means that Hypothesis 1 is supported. Time anxiety also exerted a significant positive impact on irrational procrastination (*β* = 0.52, *t* = 20.70, *p* < 0.001); and irrational procrastination exerted a significant positive impact on sleep quality (*β* = 0.10, *t* = 3.13, *p* < 0.001). These findings provided preliminary evidence, suggesting that irrational procrastination acts as a mechanism linking time anxiety to negatively affect the sleep quality of college students.

**Table 2:** Analysis of the mediating effect of irrational procrastination behavior.

Dependent variable	Independent variable	*R*	*R* ^2^	F	*β*	*t*	LLCI	ULCI
Sleep quality		0.31	0.09	29.34				
	Gender				0.11	3.53***	0.05	0.17
	Age				0.01	0.38	−0.05	0.07
	Major				−0.03	−1.05	−0.09	0.03
	Time anxiety				0.28	9.95***	0.23	0.34
Irrational procrastination		0.53	0.28	108.17				
	Gender				0.02	0.82	−0.03	0.08
	Age				−0.02	−0.86	−0.07	0.03
	Major				−0.04	−1.29	−0.09	0.02
	Time anxiety				0.52	20.70***	0.48	0.57
Sleep quality		0.32	0.10	25.61				
	Gender				0.11	3.46***	0.05	0.17
	Age				0.01	0.46	−0.04	0.07
	Major				−0.03	−0.94	−0.09	0.03
	Irrational procrastination				0.10	3.13***	0.04	0.17
	Time anxiety				0.23	6.87***	0.16	0.29

All variables in the model are substituted with standardized data, the same blow.

In addition, Bootstrap analysis was conducted to test the mediation paths. The results, as shown in Table 3, indicated that in terms of direct effects, the 95% confidence interval of time anxiety on sleep quality has a lower limit of 0.16 and an upper limit of 0.29, both of which exclude 0. This indicated that time anxiety can directly influence sleep quality, with an effect value of 0.23, accounting for 80.71% of the total direct effect. The 95% confidence interval of the mediating effect of irrational procrastination ranges from 0.02 to 0.09, and it also does not include 0. This indicated that irrational procrastination acts as a mediator between time anxiety and sleep quality, with an effect value of 0.05, accounting for 19.26% of the total mediating effect. The establishment of the mediating effect demonstrated that time anxiety not only directly and positively influences the sleep quality level of college students, but also indirectly influences it through irrational procrastination. Therefore, Hypothesis 2 is supported.

**Table 3:** Bootstrap analysis for the significance test of the mediating effect.

Effect paths	Effect size	Boot	Boot CI	Boot CI	Proportion of relative effect/%
		Standard error	Lower limit	Upper limit	
Time anxiety → Sleep quality	0.23	0.03	0.16	0.29	80.71%
Time anxiety → Irrational Procrastination → Sleep quality	0.05	0.02	0.02	0.09	19.26%
Total effect	0.28	0.03	0.23	0.34	100%

### 3.4 Test of moderated mediation model

**Figure 2:**
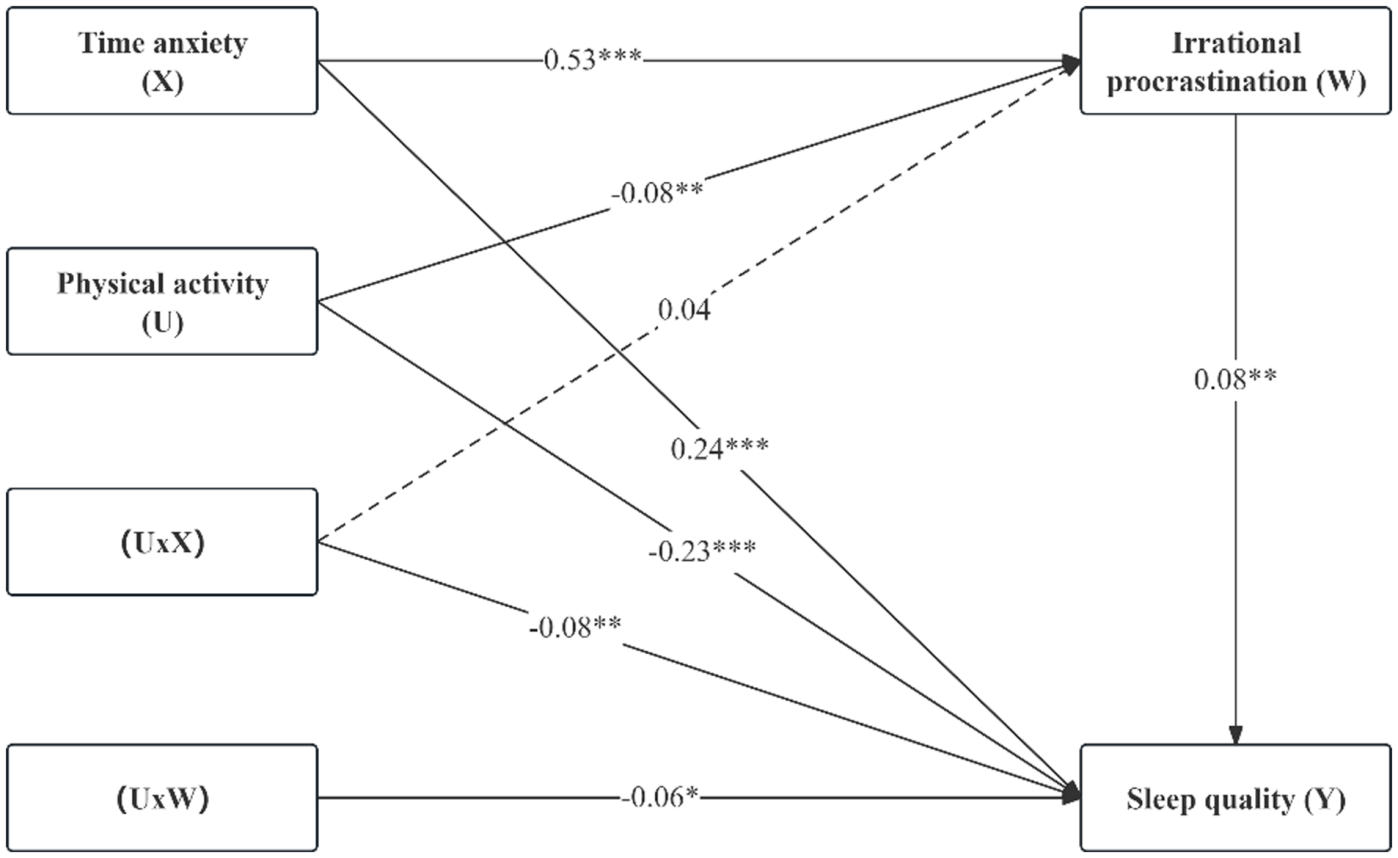
Moderated mediation model. *p < 0.05, **p < 0.01, ***p < 0.001.

In terms of moderating effect (Table 4 and Figure 2), the moderated mediation model was tested by using the PROCESS plug-in with Model 59, while controlling for gender, age, and major. After incorporating physical activity into the moderated mediation model, the interaction between time anxiety and physical activity had a negative but not significant impact on irrational procrastination (*β* = −0.04, *t* = −1.57, *p* > 0.05). The interaction between time anxiety and physical activity had a significant negative impact on sleep quality (*β =* −0.08, *t =* −2.98, *p <* 0.01). However, the interaction between irrational procrastination and physical activity had a significant negative impact on sleep quality (*β* = −0.06, *t* = −2.12, *p <* 0.05). Therefore, Hypothesis 3 is partially validated.

**Table 4:** Test of moderated mediation model.

Dependent variable	Independent variable	*R*	*R* ^2^	F	*β*	*t*	LLCI	ULCI
Irrational procrastination		0.53	0.28	74.36				
	Gender				0.01	0.18	−0.05	0.06
	Age				−0.02	−0.69	−0.07	0.03
	Major				−0.03	−1.10	−0.09	0.02
	Physical activity				−0.08	−2.95**	−0.13	−0.03
	Time anxiety				0.53	20.27***	0.47	0.58
	Time anxiety × Physical activity				−0.04	−1.57	−0.09	0.01
Sleep quality		0.40	0.16	26.34				
	Gender				0.06	1.83	0.00	0.12
	Age				0.02	0.81	−0.03	0.08
	Major				−0.01	−0.48	−0.07	0.05
	Irrational procrastination				0.08	2.62**	0.02	0.15
	Physical activity				−0.23	−7.88***	−0.29	−0.17
	Time anxiety				0.24	7.22***	0.17	0.30
	Time anxiety × Physical activity				−0.08	−2.98**	−0.14	−0.02
	Irrational procrastination × Physical activity				−0.06	−2.12*	−0.12	0.00

**Figure 3:**
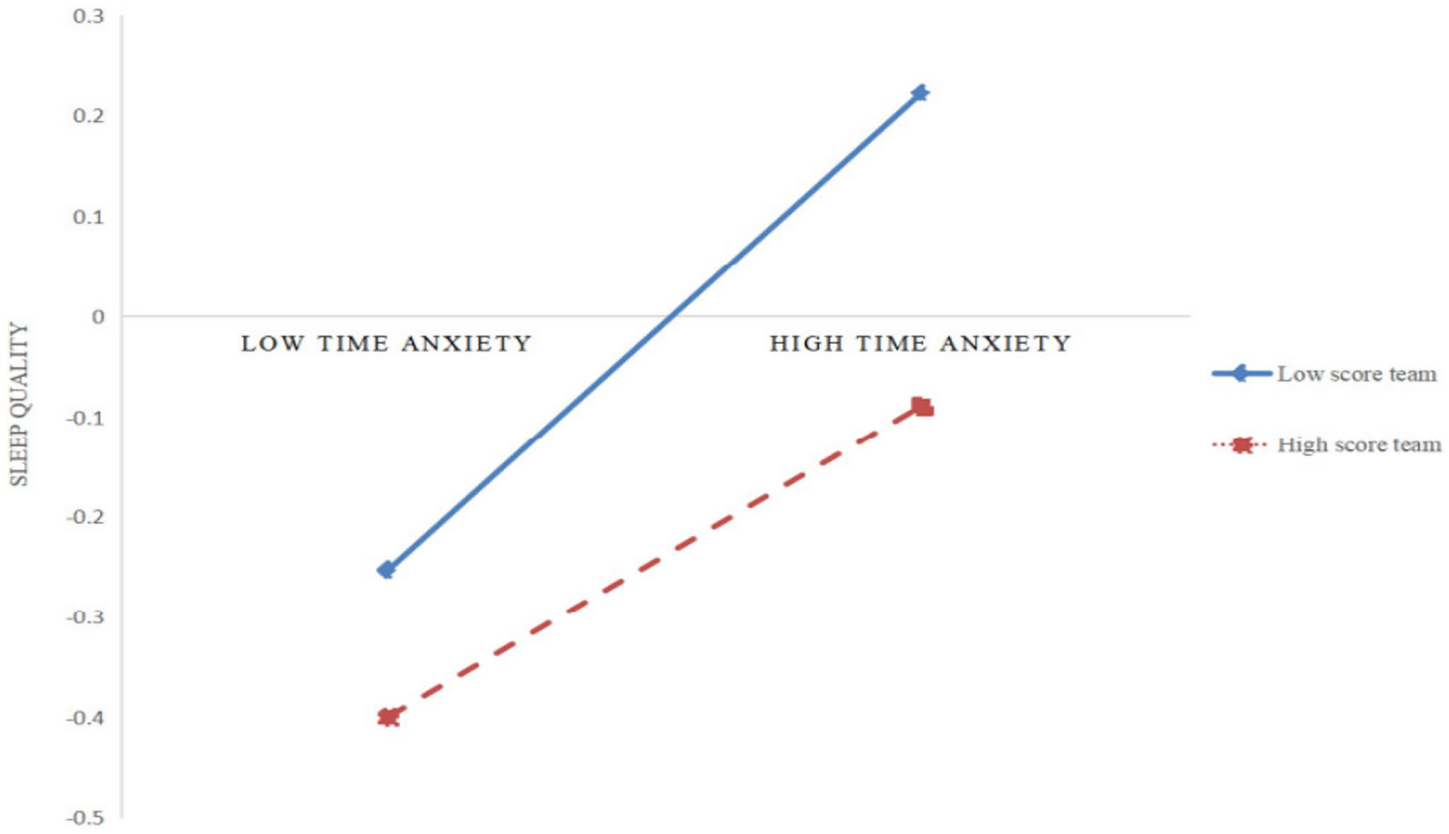
The moderating effect physical activity in the relationship between time anxiety and sleep quality.

**Figure 4:**
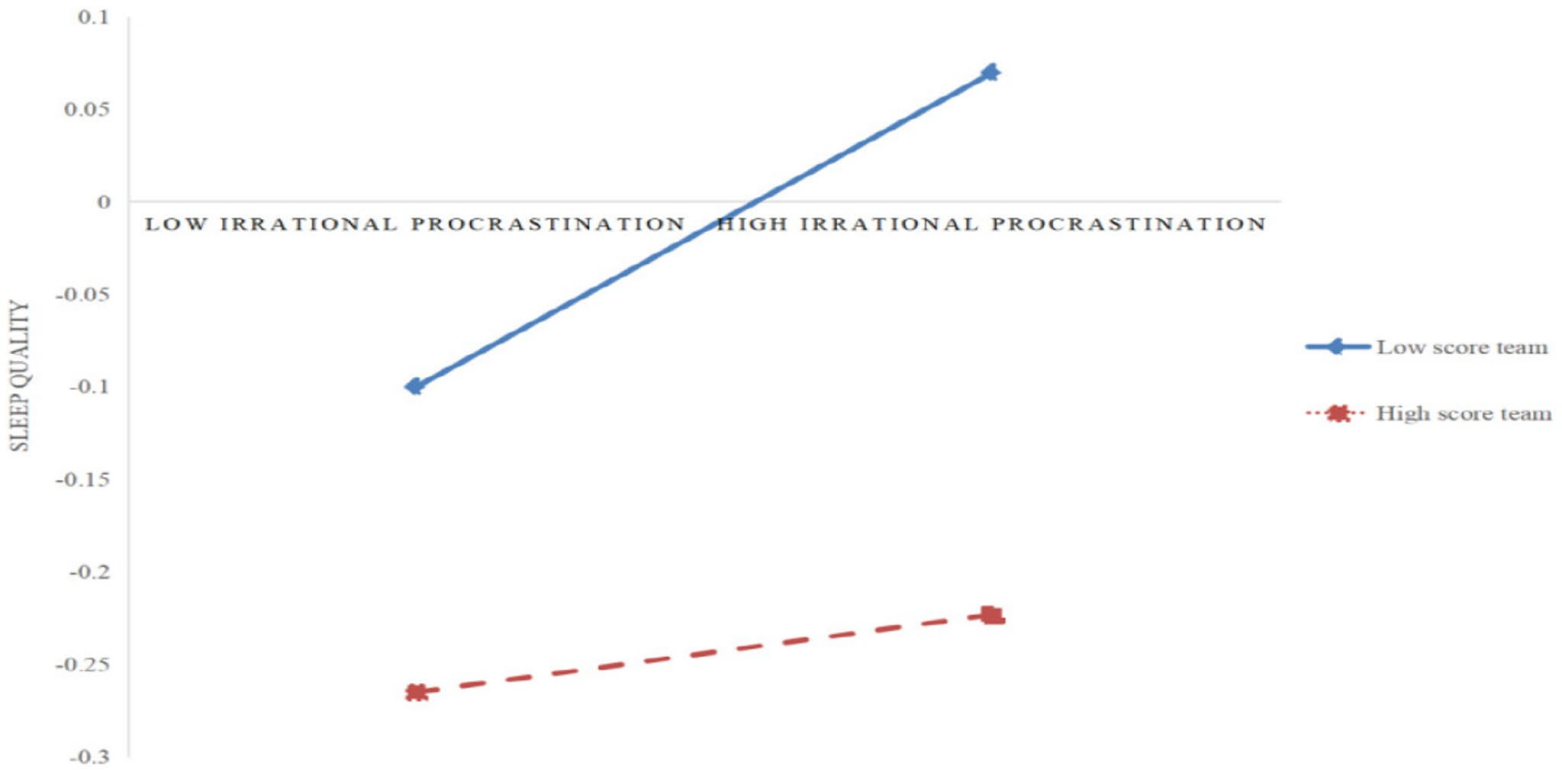
The moderating effect of physical activity in the relationship between irrational procrastination and sleep quality.

Further analysis of the paths with significant moderating effects using simple slope analysis (as shown in Figures 3, 4) revealed that for college students with low physical activity levels (*M* − 1SD), time anxiety exerted a significant positive impact on sleep quality (*simple slope* = 0.32, *t* = 6.47, *p* < 0.001), and irrational procrastination exerted a significant positive impact on sleep quality (*simple slope* = 0.15, *t* = 3.20, *p* < 0.01). However, for college students with high physical activity levels (*M* + 1SD), although time anxiety still exerted a significant positive impact on sleep quality (*simple slope* = 0.16, *t* = 3.86, *p* < 0.001), its impact was relatively diminished; but irrational procrastination exerted a relatively weak impact on sleep quality (*simple slope* = 0.02, *t* = 0.50, *p* > 0.05). This indicates that physical activity can significantly moderate the impacts of time anxiety and irrational procrastination on sleep quality. Specifically, when the physical activity level increases, the positive impacts of time anxiety and irrational procrastination on sleep quality gradually decrease, indicating an improvement in college students’ sleep quality.

## 4 Discussion

### 4.1 Time anxiety and sleep quality

This study found that time anxiety exerted a positive impact on sleep quality, confirming Hypothesis 1. It suggests that as the time anxiety score of college students increases, their sleep quality score increases, and they have poorer sleep quality. This basically aligns with previous research findings (Brummett et al., 2006; Xiao et al., 2023). The Sleep Disturbance Process Theory proposes that individuals exhibiting symptoms of depression and anxiety are more likely to experience sleep-related issues (Gehrman et al., 2011; Oh et al., 2019). As a specific manifestation of anxiety in relation to time, time anxiety may also affect college students’ sleep quality. Specifically, after leaving their original families and embarking on relatively independent social lives, college students often have strong expectations of achievement in their life and career planning. However, in pursuing these expectations, they are easily distracted by external factors such as online games and social environments, which continually deplete their self-managed time resources (Fan, 2020). This often leads to a situation where, even though they can foresee the negative impacts of procrastination, they still struggle to complete tasks with reasonable self-discipline, thus causing constant delays in their planned schedules. However, constrained by the fixed total quantity of time available, they commonly feel the anxiety of “not having enough time.” Consequently, they resort to staying up late and similar methods to gain more personal discretionary time to complete their predetermined tasks. “Despite sleepiness, I’m reluctant to spend my time on sleeping” reflects the inner struggle experienced by many college students.

### 4.2 The mediating role of irrational procrastination

This study found that irrational procrastination plays a mediating role between time anxiety and college students’ sleep quality. Hypothesis 2 has been verified, indicating that time anxiety increases the occurrence of irrational procrastination behaviors among college students, subsequently impacting their sleep quality. These findings align with previous research results (Campbell and Bridges, 2023). On the one hand, time anxiety can negatively predict irrational procrastination behavior in college students. According to the conceptual model of procrastination, negative emotions are key factors that lead individuals to engage in procrastination-related behaviors (Song et al., 2015). When college students lack time management skills, they are usually prone to experiencing negative emotions such as anxiety (Senecal et al., 1995). These emotional changes can negatively impact their self-regulation abilities, which can easily lead to irrational procrastination behavior. From a psychological mechanism perspective, individuals with high time anxiety often experience an emotional state of anxiety and unease due to poor self-time management skills (Meng and Zheng, 2006). This negative emotion makes it challenging for individuals to think rationally and calmly when devising solutions, and consumes a substantial amount of their self-control resources (Rebetez et al., 2016), leading to self-regulation failure and triggering irrational procrastination behaviors. From the perspective of brain neural mechanisms, the emergence of anxiety emotions, such as time anxiety and trait anxiety, in individuals is associated with structural and functional abnormalities in the prefrontal-limbic system neural circuit. These abnormalities are primarily evident in brain areas such as the prefrontal cortex, hippocampus, and amygdala (Craske and Stein, 2016). Functional impairments in the prefrontal cortex (Bishop, 2007), abnormal volume in the right hippocampus (Zhang et al., 2020), and enlargement of the amygdala all can diminish an individual’s self-control ability (Feng et al., 2021), thereby leading to irrational procrastination behaviors.

In addition, irrational procrastination behavior can potentially limit an individual’s sleep time and delay their bedtime, thereby reducing the quality of sleep. Specifically, college students with a high level of irrational procrastination tend to exhibit lower levels of healthy behaviors, such as laziness, evasion, and self-imposed limitations (Ni et al., 2016). As a result, they often resort to staying up late to complete tasks they have postponed, thereby replacing the time that could have been allocated for sleep. However, this behavior can lead to an increase in circadian rhythm disruptions (Huang et al., 2023), resulting in changes to the body’s internal system for maintaining and inducing circadian rhythms, or causing the endogenous circadian rhythm to become unsynchronized with the external environment. This can easily lead to difficulties in falling asleep, maintaining sleep, and insufficient sleep duration (Zhou et al., 2021), thereby reducing the quality of sleep for college students. In summary, it can be concluded that time anxiety among college students affects sleep quality and leads to sleep disorders via the mediating role of irrational procrastination behavior.

### 4.3 The moderating effect of physical activity

This study also found that physical activity moderates the direct impact of time anxiety on the sleep quality of college students, as well as the second half path of the irrational procrastination mediation model. Thus, Hypothesis 3 is partially verified.

On one hand, physical activity regulates the relationship between time anxiety and sleep quality. As physical activity level increases, the sleep disorders triggered by time anxiety is somewhat mitigated. This is consistent with previous research findings (Ji et al., 2022). Based on the integrated model of sports performance, and in light of this study, it has been observed that college students who regularly engage in physical activities are more likely to immerse themselves in an environment that promotes physical activity and make friends with peers who have positive exercise habits and behaviors, and get support from peers. This will subtly but significantly enhance the persuasive power of college students (Dong and Mao, 2018), enabling them to get timely, positive, and healthy emotional support when confronted with time anxiety. This, in turn, allows them to reconstruct their perception of time in a more peaceful and stable manner and mitigate the sleep disturbances caused by time anxiety, thereby improving college students’ sleep quality. Furthermore, from the perspective of exercise physiology, physical activity can effectively promote the secretion and release of β-endorphins in the body while suppressing the activity of adrenaline and cortisol. It can stimulate cognitive and emotional cognition (Li et al., 2015), aid in improving cognitive functions, enhance the ability to regulate emotions (Archer et al., 2014), and strengthen the “emotional buffering” effect (Crane and Ward, 2016). Consequently, physical activity can help college students reduce the negative effects of time anxiety and improve their sleep quality.

On the other hand, physical activity moderates the relationship between irrational procrastination and sleep quality. As the physical activity level decreases, the negative impact of irrational procrastination on sleep quality decreases, thereby improving their sleep quality. According to the Theory of Planned Behavior, the more positive an individual’s attitudes towards behavior and subjective norms are, the stronger their perceived control over behavior becomes. An enhanced sense of behavioral control provides greater possibilities for mitigating non-adaptive behaviors associated with procrastination (Liu et al., 2020). Specifically, for this study, when the positive attitude and behavioral patterns towards physical exercise become habitual among college student, they will develop a more proactive approach to physical activity, and manage their exercise frequency, duration, and intensity more effectively (Wei, 2023). Consequently, it helps to increase their subjective awareness of social behaviors and boosts their monitoring capabilities for planned behaviors (Harris et al., 2020). It also improves their resistance to external distractions and their focus on current task planning (Steel, 2007), allowing for efficient time planning and management. Over time, this can effectively reduce irrational procrastination among college students, leading to improved sleep quality.

## 5 Research significance and limitations

This study further verified the intrinsic relationship between time anxiety and sleep quality of college students by constructing a moderated mediation model that involves time anxiety, irrational procrastination, physical activity, and sleep quality. Therefore, this study offers valuable insights for improving the sleep quality of college students. on the one hand, colleges and universities should enhance students’ understanding of time management principles, offer time management courses, and encourage students to use smartphones, computers, and other Internet products wisely, reducing time spent on activities like excessive watching of short videos. Providing tailored guidance and intervention can assist college students in properly balancing their leisure and study time, thereby enhancing their sense of time efficiency. Consequently, this would diminish time-related anxiety and procrastination, ultimately providing auxiliary support for enhancing the sleep quality of college students. On the other hand, colleges and universities should improve college students’ understanding of physical activities through various means, and enhance their awareness of health behavior issues by offering elective courses related to physical activity, special lectures, and knowledge and skills competitions and so on. These measures will provide a favorable environment and resources to encourage college students to engage in physical activities, and enhance protective measures against time anxiety, procrastination, and issues that affect sleep quality.

Indeed, although this study preliminarily revealed the mechanisms of time anxiety, irrational procrastination, physical activity, and sleep quality through a moderated mediation model, it had its limitations that need further improvement. On the one hand, the research data collection primarily relied on self-reported questionnaires, which might introduce method effects such as memory biases and varying interpretations of concepts. Future studies could adopt diverse data collection methods. On the other hand, the cross-sectional questionnaire design of this study only allows for identifying relationships between time anxiety, irrational procrastination, physical activity, and sleep quality, without supporting causal inferences. Future research should employ experimental or longitudinal designs to further explore these dynamics.

## 6 Conclusion

A higher level of time anxiety is associated with poorer sleep quality of college students. Time anxiety not only directly affects the sleep quality of college students, but also indirectly influences it through irrational procrastination. Physical activity can effectively adjust the impacts of time anxiety and irrational procrastination on college students’ sleep quality, and serve as a protective factor to improve college students’ sleep quality. These findings offer new insights into addressing social and psychological issues, such as time anxiety, and improving the sleep quality of college students.

## Data availability statement

The original contributions presented in the study are included in the article/supplementary material, further inquiries can be directed to the corresponding author.

## Ethics statement

The studies involving humans were approved by Science and Technology Division of Yuncheng Vocational and Technical University. The studies were conducted in accordance with the local legislation and institutional requirements. The participants provided their written informed consent to participate in this study. Written informed consent was obtained from the individual(s) for the publication of any potentially identifiable images or data included in this article.

## Author contributions

ZS: Funding acquisition, Investigation, Methodology, Writing – original draft, Writing – review & editing. XG: Investigation, Software, Writing – review & editing. PR: Investigation, Writing – review & editing.

## References

[ref1] Adelantado-Renau M. Diez-Fernandez A. Beltran-Valls M. R. Soriano-Maldonado A. Moliner-Urdiales D. (2019). The effect of sleep quality on academic performance is mediated by internet use time: DADOS study. J. Pediatr. 95, 410–418. doi: 10.1016/j.jpedp.2018.06.005, PMID: 29787701

[ref2] Ajzen I. (1991). The theory of planned behavior. Organ. Behav. Hum. Decis. Process. 50, 179–211. doi: 10.1016/0749-5978(91)90020-T

[ref3] Archer T. Josefsson T. Lindwall M. (2014). Effects of physical exercise on depressive symptoms and biomarkers in depression. CNS Neurol Disord Drug Targets 13, 1640–1653. doi: 10.2174/1871527313666141130203245 25470398

[ref4] Ar-yuwat S. Clark M. J. Hunter A. James K. S. (2013). Determinants of physical activity in primary school students using the health belief model. J. Multidiscip. Healthc. 6, 119–126. doi: 10.2147/JMDH.S40876, PMID: 23569383 PMC3615973

[ref5] Baumeister R. F. Vohs K. D. Tice D. M. (2007). The strength model of self-control. Curr. Dir. Psychol. Sci. 16, 351–355. doi: 10.1111/j.1467-8721.2007.00534.x

[ref6] Berenskoetter F. (2020). Anxiety, time, and agency. INT 12, 273–290. doi: 10.1017/S1752971920000111

[ref7] Bishop S. J. (2007). Neurocognitive mechanisms of anxiety: an integrative account. Trends Cogn. Sci. 11, 307–316. doi: 10.1016/j.tics.2007.05.008, PMID: 17553730

[ref8] Brummett B. H. Babyak M. A. Siegler I. C. Vitaliano P. P. Ballard E. L. Gwyther L. P.. (2006). Associations among perceptions of social support, negative affect, and quality of sleep in caregivers and non-caregivers. Health Psychol. 25, 220–225. doi: 10.1037/0278-6133.25.2.220 16569114

[ref9] Buysse D. J. Reynolds C. F. Monk T. H. Berman S. R. Kupfer D. J. (1989). The Pittsburgh sleep quality index: a new instrument for psychiatric practice and research. Psychiat. Res. 28, 193–213. doi: 10.1016/0165-1781(89)90047-4, PMID: 2748771

[ref10] Campbell R. L. Bridges A. J. (2023). Bedtime procrastination mediates the relation between anxiety and sleep problems. J. Clin. Psychol. 79, 803–817. doi: 10.1002/JCLP.23440, PMID: 36169391

[ref11] Chen C. K. Time urgency-“experience with Chinese characteristic” in dramatic social changes of China. Ph.D. Dissertation, Nanjing University, Nanjing, China, (2013).

[ref12] Chen S. H. Xu J. M. (2022). A critique of time anxiety in a perspective of political economy of communication. Shandong Soc. Sci. 5, 104–113. doi: 10.14112/j.cnki.37-1053/c.2022.05.024

[ref13] Crane P. J. Ward S. F. (2016). Self-healing and self-Care for Nurses. AORN J. 104, 386–400. doi: 10.1016/j.aorn.2016.09.007 27793249

[ref14] Craske M. G. Stein M. B. (2016). Anxiety. Lancet 388, 3048–3059. doi: 10.1016/S0140-6736(16)30381-6 27349358

[ref15] DeSousa M. Reeve C. L. Peterman A. H. (2020). Development and initial validation of the perceived scarcity scale. Stress. Health 36, 131–146. doi: 10.1002/smi.2908, PMID: 31692256

[ref16] Dong B. L. Mao L. J. (2018). Core beliefs, deliberate rumination and exercise adherence of undergraduate: the moderated mediating effect of exercise atmosphere. J. Tianjin Univ. Sport 33, 441–447. doi: 10.13297/j.cnki.issn1005-0000.2018.05.011

[ref17] Du L. Z. Chen Y. X. (2023). Does time stressor promote or inhibit employees’ proactive work behavior? Cognitive appraisals of stress as mediator and time management skills as moderator. Hum. Res. Dev. China 40, 6–20. doi: 10.16471/j.cnki.11-2822/c.2023.4.001

[ref18] Fan Y. C. (2020). Influence of internet addiction disorder on Undergraduates' physical activities: a moderating effect of perceived social support. J. Tianjin Univ. Sport 35, 423–427+459. doi: 10.13297/j.cnki.issn1005-0000.2020.04.009

[ref19] Feng T. Y. Wang X. K. Su T. (2021). Developmental cognitive mechanism and neural basis of procrastination. Adv. Psychol. Sci. 29, 586–596. doi: 10.3724/SP.J.1042.2021.00586

[ref21] Ferrari J. R. Johnson J. L. McCown W. G. Flett G. L. Blankstein K. R. Martin T. R.. (1995). “Procrastination, negative self-evaluation, and stress in depression and anxiety: a review and preliminary model” Procrastination and task avoidance. 137–167. doi: 10.1007/978-1-4899-0227-67

[ref20] Finley A. J. Brandon J. S. (2019). Aftereffects of self-control on positive emotional reactivity. Pers. Soc. Psychol. B 45, 1011–1027. doi: 10.1177/0146167218802836, PMID: 30400747

[ref22] Gehrman P. R. Meltzer L. J. Moore M. Pack A. I. Perlis M. L. Eaves L. J.. (2011). Heritability of insomnia symptoms in youth and their relationship to depression and anxiety. Sleep 34, 1641–1646. doi: 10.5665/sleep.1424, PMID: 22131600 PMC3208840

[ref23] Gu Y. L. (2023). On time anxiety of the youth. Stud. Social. Chin. Charact. 170, 102–109.

[ref24] Hairston I. S. Shpitalni R. (2016). Procrastination is linked with insomnia symptoms: the moderating role of morningness-eveningness. Pers. Indiv. Differ. 101, 50–56. doi: 10.1016/j.paid.2016.05.031

[ref25] Harris K. Haddock G. Peters S. Gooding P. (2020). Psychological resilience to suicidal thoughts and behaviours in people with schizophrenia diagnoses: a systematic literature review. Psychol. Psychother. 93, 777–809. doi: 10.1111/papt.12255, PMID: 31625283

[ref26] Huang H. M. Sang Z. Q. (2018). Time anxiety of college students in the era of “we-media”. Jiangsu Higher Educ. 8, 96–99. doi: 10.13236/j.cnki.jshe.2018.08.020

[ref27] Huang J. H. Zhu Y. Y. Li S. Q. (2023). Relationship among daytime sleepiness, bedtime procrastination and sleep quality in college students. Chin. Ment. Health J. 37, 1065–1070.

[ref28] Ji C. Yang J. Lin L. Chen S. (2022). Physical exercise ameliorates anxiety, depression and sleep quality in college students: experimental evidence from exercise intensity and frequency. Behav. Sci. 12:61. doi: 10.3390/BS12030061, PMID: 35323380 PMC8944991

[ref30] Knowlden A. P. Naher S. (2023). Time management behavior structural equation model predicts global sleep quality in traditional entry university students. Am. J. Health 54, 265–274. doi: 10.1080/19325037.2023.2209617, PMID: 37771600 PMC10538951

[ref31] Lay C. H. (1986). At last, my research article on procrastination. J. Res. Pers. 20, 474–495. doi: 10.1016/0092-6566(86)90127-3

[ref32] Leyland A. Rowse G. Emerson L. M. (2019). Experimental effects of mindfulness inductions on self-regulation: systematic review and meta-analysis. Emotion 19, 108–122. doi: 10.1037/emo0000425, PMID: 29578742

[ref33] Li Y. Q. (2023). The emergence of “Qingjiao”: a study of time anxiety among young university teachers. Jiangsu High. Educ. 9, 96–103. doi: 10.13236/j.cnki.jshe.2023.09.012

[ref34] Li C. J. Jia H. N. Zuo J. N. (2015). Effects, mechanisms and prospects of exercise promoting mental health. China Sport Sci. Technol. 51, 132–139. doi: 10.16470/j.csst.2015.01.016

[ref35] Lian S. L. Liu Q. Q. Sun X. J. Zhou Z. K. (2018). Mobile phone addiction and college Students' procrastination: analysis of a moderated mediation mode. Psychol. Dev. Educ. 34, 595–604. doi: 10.16187/j.cnki.issn1001-4918.2018.05.10

[ref36] Liang D. Q. (1994). The relationship between stress level and physical activity of college students. Chin Mental Health J. 8, 5–6.

[ref37] Lindsay E. K. Creswell J. D. (2017). Mechanisms of mindfulness training: monitor and acceptance theory (MAT). Clin. Psychol. Rev. 51, 48–59. doi: 10.1016/j.cpr.2016.10.011, PMID: 27835764 PMC5195874

[ref38] Liu Q. (2020). Correlation between ruminant thinking and sleep quality of college students. Modern Commun. 15, 176–177.

[ref39] Liu B. L. Ke Q. Lu Y. F. (2021). The relationship between perceived stress, state-trait anxiety, and sleep quality among university graduates in China during the COVID-19 pandemic. Front. Psychol. 12:664780. doi: 10.3389/FPSYG.2021.664780, PMID: 34603119 PMC8484744

[ref40] Liu L. Shi Y. (2009). The definition, state and developing trend of clinical sport psychology. China Sport Sci. Technol. 45, 76–82. doi: 10.16470/j.csst.2009.06.007

[ref41] Liu G. R. Teng X. Q. Wang M. Q. (2020). The relationship among perfectionism, academic procrastination and cell phone addiction of college students: a mediated model. Chin. J. Spec. Educ. 9, 88–96.

[ref42] Lundh L. G. Broman J. E. (2000). Insomnia as an interaction between sleep-interfering and sleep-interpreting processes. J. Psychosom. Res. 49, 299–310. doi: 10.1016/S0022-3999(00)00150-1 11164054

[ref43] Lutz J. Bruhl A. B. Scheerer H. Jancke L. Herwig U. (2016). Neural correlates of mindful self-awareness in mindfulness meditators and meditation-naïve subjects revisited. Biol. Psychol. 119, 21–30. doi: 10.1016/j.biopsycho.2016.06.010, PMID: 27377788

[ref44] Maheshwari G. Shaukat F. (2019). Impact of poor sleep quality on the academic performance of medical students. Cureus J. Med. Sci. 11:e4357. doi: 10.7759/cureus.4357, PMID: 31192062 PMC6550515

[ref45] Meng Q. Zheng Y. (2006). Review on the research of procrastination. J. Southwest Univ. 4, 9–12. doi: 10.13718/j.cnki.xdsk.2006.04.003

[ref46] Ni S. G. Xu J. H. Ye L. (2016). Revision of irrational procrastination scale in Chinese college students and its relations with self-efficacy and health behavior. Chin. J. Clin. Psych. 101, 50–56. doi: 10.16128/j.cnki.1005-3611.2012.05.005

[ref47] Oh C. M. Kim H. Y. Na H. K. Cho K. H. (2019). The effect of anxiety and depression on sleep quality of individuals with high risk for insomnia: a population-based study. Front. Neurol. 10:849. doi: 10.3389/fneur.2019.00849, PMID: 31456736 PMC6700255

[ref48] Peng H. J. Wan L. H. Huang Y. Y. Deng S. F. Gao L. L. (2012). Investigation on health beliefs and health behaviors of stroke patients. Chin. J. Nurs. 47, 10–13.

[ref49] Pychyl T. A. Flett G. L. (2012). Procrastination and self-regulatory failure: an introduction to the special issue. J. Ration. Emot. Cogn. B 30, 203–212. doi: 10.1007/s10942-012-0149-5

[ref50] Rebetez M. M. L. Rochat L. Barsics C. Van der Linden M. (2016). Procrastination as a self-regulation failure: the role of inhibition, negative affect, and gender. Pers. Individ. Dif. 101, 435–439. doi: 10.1016/j.paid.2016.06.049

[ref52] San Román-Mata S. Puertas-Molero P. Ubago-Jiménez J. L. González-Valero G. (2020). Benefits of physical activity and its associations with resilience, emotional intelligence, and psychological distress in university students from southern Spain. Int. J. Env. Res. Pub. He. 17:4474. doi: 10.3390/ijerph17124474 PMC7344387 32580322

[ref53] Scovelle A. J. Hewitt B. Lallukka T. O'Neil A. King T. L. (2023). Time use, time pressure and sleep: is gender an effect modifier? Eur. J. Pub. Health 33, 411–417. doi: 10.1093/EURPUB/CKAD038, PMID: 36940672 PMC10234670

[ref54] Senecal C. Koestner R. Vallerand R. J. (1995). Self-regulation and academic procrastination. J. Soc. Psychol. 135, 607–619. doi: 10.1080/00224545.1995.9712234

[ref55] Sirois F. M. (2007). “I’ll look after my health, later”: a replication and extension of the procrastination–health model with community-dwelling adults. Pers. Individ. Dif. 43, 15–26. doi: 10.1016/j.paid.2006.11.003

[ref56] Sirois F. M. Melia-Gordon M. L. Pychyl T. A. (2003). “I'll look after my health, later”: an investigation of procrastination and health. Pers. Individ. Dif. 35, 1167–1184. doi: 10.1016/S0191-8869(02)00326-4

[ref57] Song M. Su T. Feng T. (2015). The model of procrastination on time orientation. Adv. Psychol. Sci. 23, 1216–1225. doi: 10.3724/SP.J.1042.2015.01216

[ref58] Steel P. (2007). The nature of procrastination: a meta-analytic and theoretical review of quintessential self-regulatory failure. Psychol. Bull. 133, 65–94. doi: 10.1037/0033-2909.133.1.65, PMID: 17201571

[ref59] Steel P. (2010). Arousal, avoidant and decisional procrastinators: do they exist? Pers. Individ. Dif. 48, 926–934. doi: 10.1016/j.paid.2010.02.025

[ref60] Sun L. Li K. Q. Zhang Y. S. Zhang L. L. (2021). Differentiating the associations between sleep quality and suicide behaviors: a population-based study in China. J Affect. Disorders. 297, 553–558. doi: 10.1016/J.JAD.2021.10.126, PMID: 34728292

[ref61] Tao S. M. Wu X. Y. Zhang S. C. Tong S. L. Hao J. H. Tao F. B. (2017). Association of alcohol use with problematic mobile phone use and depressive symptoms among college students in Anhui. China. J. Public Health UK 25, 103–112. doi: 10.1007/s10389-016-0766-z

[ref62] Tillich P. (2000). The collected works of Paul Tillich (Volume II). Shanghai: Shanghai Sanlian Publishing House.

[ref63] Torre R. R. (2007). Time's social metaphors: an empirical research. Time Soc. 16, 157–187. doi: 10.1177/0961463X07080262

[ref64] Usunier J. C. G. Pierre V. F. (1994). Perceptual time patterns (time-Styles') a psychometric scale. Time Soc. 3, 219–241. doi: 10.1177/0961463X94003002005

[ref65] Verhaeghen P. (2021). Mindfulness as attention training: Meta-analyses on the links between attention performance and mindfulness interventions, long-term meditation practice, and trait mindfulness. Mindfulness 12, 564–581. doi: 10.1007/s12671-020-01532-1

[ref66] Wei Z. F. (2023). Relationship between physical exercise and academic procrastination of college students: a chain mediation of self-control and Mobile phone dependence. J. Sheny. Sport Univers. 42, 24–32.

[ref67] Wu W. Yao H. B. Zhang R. (2014). The relationship between medical Students' self-efficacy, self-esteem and anxiety: the mediating effect of time management disposition. Chin. J. Health Stat. 31, 749–751+755.

[ref68] Xi Y. B. (2004). The concept of physical exercise and its methodology. J. Beij. Sport Univers. 1, 118–120. doi: 10.19582/j.cnki.11-3785/g8.2004.01.045

[ref69] Xiao H. Shen Y. L. Zhang W. Z. Lin R. M. (2023). Applicability of the cognitive model of generalized anxiety disorder to adolescents’ sleep quality: a cross-sectional and longitudinal analysis. Int. J. Clin. Hlth. Psyc. 23:100406. doi: 10.1016/J.IJCHP.2023.100406, PMID: 37663041 PMC10472235

[ref70] Xu W. (2020). Trait mindfulness: a new visual angle for the study of exercise psychology. J. Chengdu Sport Univers. 46, 94–99. doi: 10.15942/j.jcsu.2020.01.015

[ref001] Yang J. Y. Li Y. F. (2023). Exercise Improving Sleep: Current Status and Reflections on PSG-based Research. J. Capital Univ. Phys. Educ. Sports. 35, 475–482. doi: 10.14036/j.cnki.cn11-4513.2023.05.002

[ref71] You Z. Q. Mei W. J. Ye N. Zhang L. Andrasik F. (2021). Mediating effects of rumination and bedtime procrastination on the relationship between internet addiction and poor sleep quality. J. Behav. Addict. 9, 1002–1010. doi: 10.1556/2006.2020.00104, PMID: 33399544 PMC8969718

[ref72] Zhang R. Chen Z. Xu T. Zhang L. Feng T. (2020). The overlapping region in right hippocampus accounting for the link between trait anxiety and procrastination. Neuropsychologia 146:107571. doi: 10.1016/j.neuropsychologia.2020.107571, PMID: 32721496

[ref73] Zheng Z. G. Liu J. P. Dong S. H. Jiang Y. Liao H. (2018). The relationship between high grade Pupils' parenting styles and academic procrastination: the mediating effect of time management disposition. Stud. Psychol. Behav. 16, 786–792.

[ref74] Zhou X. H. (2014). Anxiety: the symptom of the times under the background of rapid changes. J. Jiangsu Administ. Instit. 78, 54–57.

[ref75] Zhou K. L. Du X. Y. Wan Y. H. Zhang X. Gao W. Tao H. Y.. (2021). Study on the changes of neuropsychology and sleep microstructure in patients with circadian rhythm disorder sleep-wake disorder with depression and anxiety treated by transcranial magnetic stimulation. Chin. J. Rehabilit. Med. 36, 1287–1291. doi: 10.27366/d.cnki.gtyku.2019.000648

[ref76] Zhu Y. Y. Huang J. H. (2022). The relationship between bedtime procrastination and daytime sleepiness in college students: a moderated mediation model. Stud. Psychol. Behav. 20, 797–804.

